# Complete genome sequence of *Capnocytophaga ochracea* type strain (VPI 2845^T^)

**DOI:** 10.4056/sigs.15195

**Published:** 2009-09-24

**Authors:** Konstantinos Mavrommatis, Sabine Gronow, Elizabeth Saunders, Miriam Land, Alla Lapidus, Alex Copeland, Tijana Glavina Del Rio, Matt Nolan, Susan Lucas, Feng Chen, Hope Tice, Jan-Fang Cheng, David Bruce, Lynne Goodwin, Sam Pitluck, Amrita Pati, Natalia Ivanova, Amy Chen, Krishna Palaniappan, Patrick Chain, Loren Hauser, Yun-Juan Chang, Cynthia D. Jeffries, Thomas Brettin, John C. Detter, Cliff Han, James Bristow, Markus Göker, Manfred Rohde, Jonathan A. Eisen, Victor Markowitz, Nikos C. Kyrpides, Hans-Peter Klenk, Philip Hugenholtz

**Affiliations:** 1 DOE Joint Genome Institute, Walnut Creek, California, USA; 2 DSMZ - German Collection of Microorganisms and Cell Cultures GmbH, Braunschweig, Germany; 3 Los Alamos National Laboratory, Bioscience Division, Los Alamos, New Mexico, USA; 4 Oak Ridge National Laboratory, Oak Ridge, Tennessee, USA; 5 Biological Data Management and Technology Center, Lawrence Berkeley National Laboratory, Berkeley, California, USA; 6 Lawrence Livermore National Laboratory, Livermore, California, USA; 7 HZI - Helmholtz Centre for Infection Research, Braunschweig, Germany; 8 University of California Davis Genome Center, Davis, California, USA

**Keywords:** gliding, capnophilic, periodontitis, gingivitis, *Flavobacteriaceae*

## Abstract

*Capnocytophaga ochracea* (Prévot et al. 1956) Leadbetter et al. 1982 is the type species of the genus *Capnocytophaga*. It is of interest because of its location in the *Flavobacteriaceae*, a genomically not yet charted family within the order *Flavobacteriales*. The species grows as fusiform to rod shaped cells which tend to form clumps and are able to move by gliding. *C. ochracea* is known as a capnophilic (CO_2_-requiring) organism with the ability to grow under anaerobic as well as aerobic conditions (oxygen concentration larger than 15%), here only in the presence of 5% CO_2_. Strain VPI 2845^T^, the type strain of the species, is portrayed in this report as a gliding, Gram-negative bacterium, originally isolated from a human oral cavity. Here we describe the features of this organism, together with the complete genome sequence, and annotation. This is the first completed genome sequence from the flavobacterial genus *Capnocytophaga*, and the 2,612,925 bp long single replicon genome with its 2193 protein-coding and 59 RNA genes is a part of the *** G****enomic* *** E****ncyclopedia of* *** B****acteria and* *** A****rchaea * project.

## Introduction

Strain VPI 2845^T^ (= DSM 7271 = ATCC 27872 =JCM 1296) is the type strain of *Capnocytophaga ochracea*, and the type species of the genus *Capnocytophaga. C. ochracea* was first described by Prévot *et al.* [1] as ‘*Fusiformis nucleatus* var. *ochraceus’* and later renamed by Leadbetter *et al* [2]. Other synonyms for *C. ochracea* are *'Bacteroides oralis* var. *elongatus'* [3],*'Bacteroides ochraceus'* (basonym) [4] and "*Ristella ochraceus*" (*sic*) [5]. The organism is of significant interest for its position in the tree of life where the genus *Capnocytophaga* (8 species) is located within the large family of the *Flavobacteriaceae*. First, Leadbetter *et al.* placed the genus *Capnocytophaga* in the family of the *Cytophagaceae* within the order *Cytophagales* [6] which was emended in 2002 by the Subcommittee on the Taxonomy of *Flavobacterium* and *Cytophaga*-like bacteria of the International Committee on Systematics of Prokaryotes [7]. *C. ochracea* is most often found in association with animal and human hosts. In general, it is a normal inhabitant of the human mouth and other non-oral sites. *C. ochracea* is associated with juvenile and adult periodontitis [8,9] and may cause severe infections in immunocompromised as well as in immunocompetent patients [10-12]. Among these are endocarditis, endometritis, osteomyelitis, abscesses, peritonitis, and keratitis. Here we present a summary classification and a set of features for *C. ochracea* VPI 2845^T^ together with the description of the complete genomic sequence and annotation.

## Classification and features

Genbank lists 16S rRNA sequences for only a few small number of cultivated strains belonging to *C. ochraceae*, all of them isolated from human oral cavity(e.g. U41351, U41353, DQ012332). Phylotypes (sequences from uncultivated bacteria) closely linked to *C. ochracea* also originate in almost exclusively from human oral samples collected from European, American, Asian and African samples (AF543292, AF543298, AY278613, AM420149, AY429469, FJ470418). Only two bacterial clones are reported from non-human sources. One was isolated from *Strongylocentrotus intermedius* (sea urchin) in the Sea of Japan (EU432412, EU432438), and the second from *Oncorhynchus mykiss* (rainbow trout) caught in Scotland (AM179907). Screening of environmental genomic samples and surveys reported at the NCBI BLAST server indicated no closely related phylotypes (>91% sequence identity) that can be linked to the species or genus.

**Figure 1:**
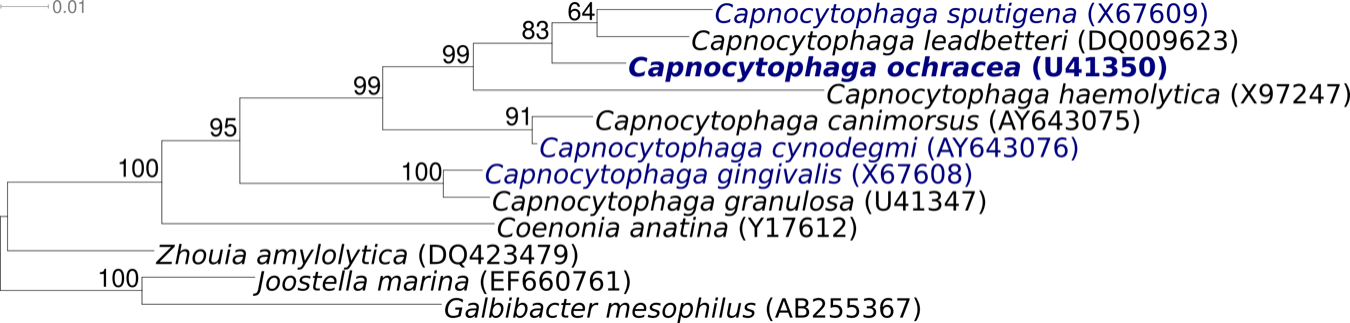
Phylogenetic tree highlighting the position of *C. ochracea* VP 2845^T^ relative to the other type strains of species within the genus *Capnocytophaga* and to selected type strains of species belonging to other genera within the *Flavobacteriaceae*. The tree was inferred from 1,405 aligned characters [13,14] of the 16S rRNA gene sequence under the maximum likelihood criterion [15] and rooted with *Joostella* and *Galbibacter*. The branches are scaled in terms of the expected number of substitutions per site. Numbers above branches are support values from 1,000 bootstrap replicates if larger than 60%. Lineages with type strain genome sequencing projects registered in GOLD [16] are shown in blue, published genomes in bold.

Figure 1 shows the phylogenetic neighborhood of *C. ocharcea* VPI 2845^T^ in a 16S rRNA based tree. All four 16S rRNA gene copies in the genome of strain VPI 2845^T^ are identical, but differ by two nucleotides from the previously published 16S rRNA sequence (U41350) generated from ATCC 27872.

**Figure 2:**
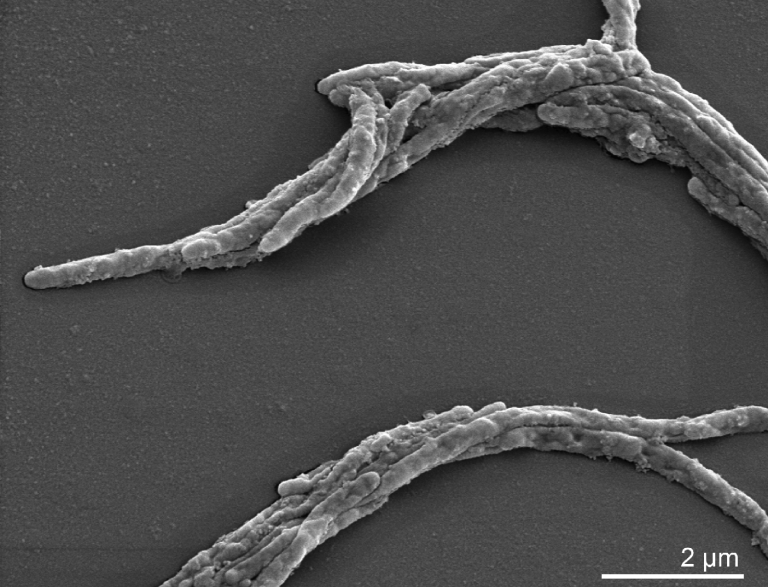
Scanning electron micrograph of *C. ochracea* VPI 2845^T^

*C. ochracea* is Gram-negative, has no flagellae and is motile by gliding (Table 1, Figure 2). Cells are pigmented and the name ‘ochracea’ is derived from the yellow color exhibited by harvested cell mass [6]. It is a catalase- and oxidase-negative species. *C. ochracea* is usually susceptible to a number of antibiotics, however, resistance is increasing in this species [23,24]. Furthermore, *C. ochracea* is known to possess an immunosuppressive factor [25]. All strains of *C. ochracea* are capable of fermenting glucose, sucrose, maltose and mannose, whereas most strains ferment amygdalin, fructose, galactose, lactose and raffinose [20]. The optimal growth temperature is 37°C. Nitrate is reduced to nitrite, and dextran, glycogen, starch and aesculin are hydrolysed by most strains. Indole is not produced. Acetic and succinic acid are the main metabolic end products of fermentation [6].

**Table 1:** Classification and general features of *C. ochracea* VPI 2845^T^ in accordance to the MIGS recommendations [17]

**MIGS ID**	**Property**	**Term**	**Evidence code**
	Current classification	Domain *Bacteria*	TAS [18]
		Phylum *‘Bacteroidetes’*	TAS [19]
		Class *Flavobacteria*	TAS [19]
		Order *Flavobacteriales*	TAS [7]
		Family *Flavobacteriaceae*	TAS [7]
		Genus *Capnocytophaga*	TAS [6]
		Species *Capnocytophaga ochracea*	TAS [6]
		Type strain VPI 2845	TAS [6]
	Gram stain	negative	TAS [6]
	Cell shape	fusiform rods	TAS [6]
	Motility	gliding	TAS [6]
	Sporulation	non-sporulating	TAS [6]
	Temperature range	mesophile	NAS
	Optimum temperature	30-37°C	NAS
	Salinity	nonhalophile	NAS
MIGS-22	Oxygen requirement	capnophilic; aerobic or anaerobic with at least 5% CO_2_	TAS [6]
	Carbon source	glucose, maltose, lactose, sucrose	TAS [20]
	Energy source	chemoorganotroph, carbohydrates	NAS
MIGS-6	Habitat	human oral cavity	TAS [3]
MIGS-15	Biotic relationship	unknown	NAS
MIGS-14	Pathogenicity	opportunistic pathogen	TAS [12]
	Biosafety level	2	TAS [21]
	Isolation	human oral cavity	TAS [2]
MIGS-4	Geographic location	not reported	
MIGS-5	Sample collection time	not reported	
MIGS-4.1 MIGS-4.2	Latitude – Longitude	not reported	
MIGS-4.3	Depth	not reported	
MIGS-4.4	Altitude	not reported	

Evidence codes - IDA: Inferred from Direct Assay (first time in publication); TAS: Traceable Author Statement (i.e., a direct report exists in the literature); NAS: Non-traceable Author Statement (i.e., not directly observed for the living, isolated sample, but based on a generally accepted property for the species, or anecdotal evidence). These evidence codes are from the Gene Ontology project [22]. If the evidence code is IDA, then the property was directly observed for a living isolate by one of the authors or an expert mentioned in the acknowledgements.

Analysis of amino acids and amino sugars of the peptidoglycan revealed that glucosamine, muramic acid, D-glutamic acid, alanine, and diaminopimelic acid were the principal components and the peptidoglycan belongs to the Alγ-type. Serine and glycine were not found [26]. As in other *Capnocytophaga* strains, the fatty acid pattern of strain *C. ochracea* VPI 2845^T^ is dominated by *iso*-branched chain saturated fatty acids i-C_15:0_ (63.5%), C_18:2_ (8.1%) and i-3OH C_17:0_ (13.8%) [23,27,28]. Phosphatidylethanolamine and an ornithine-amino lipid were identified as dominating polar lipids, as well as lesser amounts of lysophosphatidyl-ethanolamine [29]. In addition, the unusual sulfonolipid capnine (2-amino-3-hydroxy-15-methylhexadecane-1-sulfonic acid) was identified as major cell wall component [30].

## Genome sequencing and annotation

### Genome project history

This organism was selected for sequencing on the basis of its phylogenetic position, and is part of the *** G****enomic* *** E****ncyclopedia of* *** B****acteria and* *** A****rchaea * project. The genome project is deposited in the Genomes OnLine Database [10] and the complete genome sequence in GenBank (CP001632). Sequencing, finishing and annotation were performed by the DOE Joint Genome Institute (JGI). A summary of the project information is shown in Table 2.

**Table 2:** Genome sequencing project information

**MIGS ID**	**Property**	**Term**
MIGS-31	Finishing quality	Finished
MIGS-28	Libraries used	Two Sanger libraries: 6.5kb pMCL200 and fosmid pcc1Foslibraries and one 454 pyrosequence standard library
MIGS-29	Sequencing platforms	ABI3730, 454GS FLX
MIGS-31.2	Sequencing coverage	9.9× Sanger; 25.2× pyrosequence
MIGS-20	Assemblers	Newbler, phrap
MIGS-32	Gene calling method	Prodigal, GenePrimp
	INSDC / Genbank ID	CP001632
	Genbank Date of Release	August 26, 2009
	GOLD ID	Gc01027
	NCBI project ID	29403
	Database: IMG-GEBA	2501416900
MIGS -13	Source material identifier	DSM 7271
	Project relevance	Tree of Life, GEBA, Medical

### Growth conditions and DNA isolation

*C. ochracea* VPI 2845^T^, DSM 7271, was grown under anaerobic conditions in DSMZ medium 340 (*Capnocytophaga* Medium, [31]) plus 0.1% NaHCO_3_ at 37°C. DNA was isolated from 1-1.5 g of cell paste using Qiagen Genomic 500 DNA Kit (Qiagen, Hilden, Germany) with a modified protocol, L, for cell lysis, as described in Wu *et al*. [32].

### Genome sequencing and assembly

The genome was sequenced using a combination of Sanger and 454 sequencing platforms. All general aspects of library construction and sequencing performed at the JGI can be found at the JGI website. 454 Pyrosequencing reads were assembled using the Newbler assembler version 1.1.02.15 (Roche). Large Newbler contigs were broken into 2,919 overlapping fragments of 1,000 bp and entered into assembly as pseudo-reads. The sequences were assigned quality scores based on Newbler consensus q-scores with modifications to account for overlap redundancy and to adjust inflated q-scores. A hybrid 454/Sanger assembly was made using the parallel phrap assembler (High Performance Software, LLC). Possible mis-assemblies were corrected with Dupfinisher or transposon bombing of bridging clones [33]. Gaps between contigs were closed by editing in Consed, custom primer walk or PCR amplification. A total of 226 Sanger finishing reads were produced to close gaps, to resolve repetitive regions, and to raise the quality of the finished sequence. The error rate of the completed genome sequence is less than 1 in 100,000. Together all sequence types provided 35.1× coverage of the genome.

### Genome annotation

Genes were identified using Prodigal [34] as part of the Oak Ridge National Laboratory genome annotation pipeline, followed by a round of manual curation using the JGI GenePRIMP pipeline [35]. The predicted CDSs were translated and used to search the National Center for Biotechnology Information (NCBI) nonredundant database, UniProt, TIGRFam, Pfam, PRIAM, KEGG, COG, and InterPro databases. Additional gene prediction analysis and functional annotation were performed within the Integrated Microbial Genomes Expert Review (IMG-ER) platform [36].

### Genome properties

**Figure 3:**
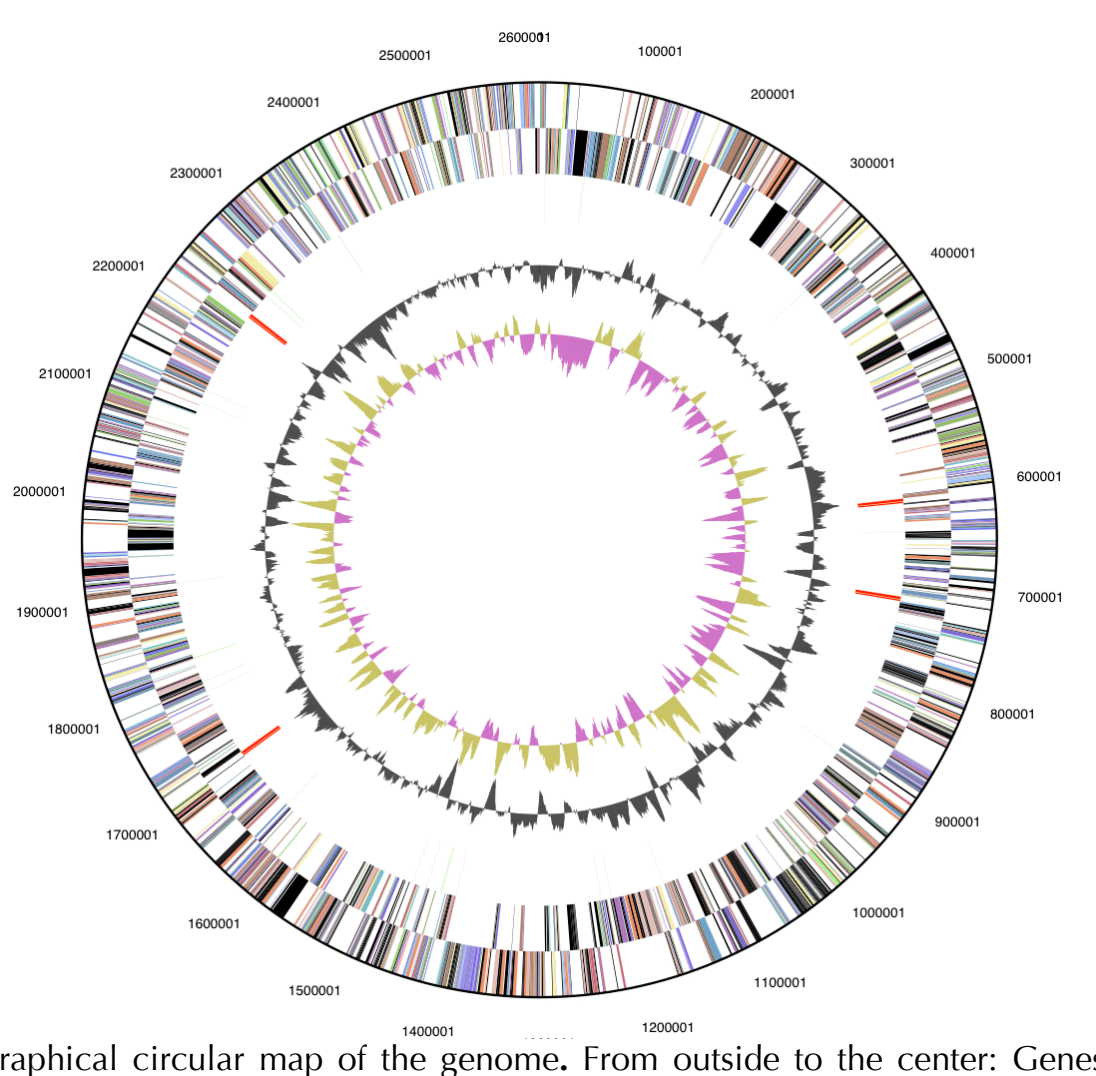
**Graphical circular map of the genome.** From outside to the center: Genes on forward strand (color by COG categories), Genes on reverse strand (color by COG categories), RNA genes (tRNAs green, rRNAs red, other RNAs black), GC content, GC skew.

The genome is 2,612,925 bp long and comprises one circular chromosome with a 39.6% GC content (Table 3). Of the 2,252 genes predicted, 2,193 were protein coding genes, and 59 RNAs; 22 pseudogenes were also identified. Genes assigned with putative functions comprised 61.7% of the genome, while the remaining genes were annotated as hypothetical proteins. The properties and the statistics of the genome are summarized in Table 3. The distribution of genes into COG functional categories is presented in Figure 3 and Table 4.

**Table 3:** Genome Statistics

**Attribute**	**Value**	**% of Total**
Genome size (bp)	2,612,925	100.00%
DNA Coding region (bp)	2,293,132	87.76%
DNA G+C content (bp)	1,034,404	39.59%
Number of replicons	1	
Extrachromosomal elements	0	
Total genes	2,252	100.00%
RNA genes	59	2.62%
rRNA operons	4	
Protein-coding genes	2,193	97.38%
Pseudo genes	22	0.98%
Genes with function prediction	1,403	62.3%
Genes in paralog clusters	207	9.19%
Genes assigned to COGs	1,330	59.06%
Genes assigned Pfam domains	1,379	61.23%
Genes with signal peptides	602	26.73%
Genes with transmembrane helices	471	20.91%
CRISPR repeats	1	

**Table 4:** Number of genes associated with the general COG functional categories

**Code**	**value**	**%age**	**Description**
J	134	6.1	Translation
A	0	0.0	RNA processing and modification
K	55	2.5	Transcription
L	83	3.8	Replication, recombination and repair
B	0	0.0	Chromatin structure and dynamics
D	19	0.9	Cell cycle control, mitosis and meiosis
Y	0	0.0	Nuclear structure
V	34	1.6	Defense mechanisms
T	35	1.6	Signal transduction mechanisms
M	158	7.2	Cell wall/membrane biogenesis
N	7	0.3	Cell motility
Z	0	0.0	Cytoskeleton
W	0	0.0	Extracellular structures
U	35	1.6	Intracellular trafficking and secretion
O	61	2.8	Posttranslational modification, protein turnover, chaperones
C	69	3.1	Energy production and conversion
G	97	4.4	Carbohydrate transport and metabolism
E	90	4.1	Amino acid transport and metabolism
F	56	2.6	Nucleotide transport and metabolism
H	84	3.8	Coenzyme transport and metabolism
I	53	2.4	Lipid transport and metabolism
P	80	3.6	Inorganic ion transport and metabolism
Q	25	1.1	Secondary metabolites biosynthesis, transport and catabolism
R	145	6.6	General function prediction only
S	100	4.6	Function unknown
-	863	39.4	Not in COGs
